# The *Shamba Chef* Educational Entertainment Program to Promote Modern Cookstoves in Kenya: Outcomes and Dose–Response Analysis

**DOI:** 10.3390/ijerph17010162

**Published:** 2019-12-25

**Authors:** W. Douglas Evans, Bonnie N Young, Michael A Johnson, Kirstie A. Jagoe, Dana Charron, Madeleine Rossanese, K Lloyd Morgan, Patricia Gichinga, Julie Ipe

**Affiliations:** 1 Milken Institute School of Public Health, The George Washington University, Washington, DC 20052, USA; 2 Colorado State University, Fort Collins, CO 80523, USA; Bonnie.Young@colostate.edu; 3 Berkeley Air Monitoring Group, Berkeley University, Berkeley, CA 94704, USA; mjohnson@berkeleyair.com (M.A.J.); kajagoe@gmail.com (K.A.J.); dcharron@berkeleyair.com (D.C.); mrossanese@berekleyair.com (M.R.); 4 Mediae Company, Nairobi 00100, Kenya; kate@mediae.org (K.L.M.); patricia@mediae.org (P.G.); 5 Clean Cooking Alliance, Washington, DC 20052, USA; jipe@cleancookstoves.org

**Keywords:** cookstoves, public health, environment, social marketing, behavior change communication, demand creation

## Abstract

Background: Globally, an estimated 3.6 billion people rely on solid fuels for cooking over open fires or in simple cookstoves. Universal access to clean cooking fuels and technology by 2030 is a United Nations’ Sustainable Development Goal. Methods: The Mediae Company created a home makeover television and radio show, *Shamba Chef*, designed to promote modern, cleaner, safer cooking methods and improved nutrition in Kenya, which reached 5 million homes in late 2017. This was accompanied by a mobile phone platform called iChef. Researchers evaluated the effects of *Shamba Chef* on cookstove purchase, use, and attitudes, beliefs, and intentions. Results: The study revealed dose–response effects of *Shamba Chef* exposure on several key outcomes. Exposure to the program was associated with an awareness of improved biomass stoves (OR 4.4; 95% CI 2.8 to 6.9), and aspirations to own an improved biomass stove (OR 2.0; 95% CI 1.4 to 2.9). Receiving information about modern stoves from two or more sources generated greater awareness of liquefied petroleum gas (LPG) stoves (OR 2.0; 95% CI 1.3 to 3.1). The qualitative study revealed that *Shamba Chef* explained how the stoves worked, communicated their benefits, and encouraged participants to trust and purchase those cookstoves. Conclusion: *Shamba Chef* was successful in influencing determinants of cookstove purchase and use, and there is evidence from the qualitative study that it influenced the purchase and use of improved biomass stoves.

## 1. Introduction

Globally, an estimated 3.6 billion people rely on solid fuels such as wood, animal dung, crop waste, coal, and charcoal for cooking over open fires or in simple cookstoves [1]. Persistent exposure to health damaging pollutants, emitted from household air pollution from cooking with solid fuels, is estimated to be responsible for 1.6 million premature deaths, and just under 60 million disability-adjusted life-years in 2017 [2]. The use of solid fuels and kerosene on rudimentary, inefficient cookstoves also increases pressures on natural resources, contributing to environmental degradation, resource depletion, and climate change through CO_2_ and black carbon emissions, as well as other hazardous pollutants [3,4]. Time spent cooking and procuring large amounts of biomass fuel can prevent the cooks, mostly women, from taking advantage of income generating and educational opportunities [1]. Furthermore, households who purchase fuel to use in traditional or rudimentary stoves often have much higher annual fuel costs than those who use more efficient technologies [5]. On the other hand, more efficient cooking technologies have the potential to make a substantial contribution to income and other economic activity, while cleaner alternatives have the potential to reduce emissions, reducing health and climate impacts [6]. Modern cooking options include well-engineered biomass stoves, electric stoves, or stoves using liquefied petroleum gas (LPG), natural gas, ethanol, and in some cases, pellets, briquettes or other compressed biomass fuels. The United Nations has set Sustainable Development Goals (SDGs) which include universal access to affordable, reliable, sustainable and modern energy—including for cooking—by 2030 [7].

In recent years, behavior change communication (BCC) and social marketing have begun to be applied to the challenge of increasing awareness, uptake and use of modern cooking options [8]. These strategies have the potential to reach large audiences on a national and regional scale and generate levels of demand that can spur commercial scale-up of these technologies and fuels. Similar BCC approaches have been successfully applied in other public health sectors, resulting in targeting of socially marketed product subsidies following a process that has been termed the Total Market Approach (TMA) [9,10].

As part of a larger project supported by the Clean Cooking Alliance (The Alliance), funded by the United Kingdom Department of Foreign Investment and Development (DFID), and elsewhere [8], the Mediae Company (Mediae)—an educational entertainment producer—created the home makeover television and radio show, *Shamba Chef*, designed to promote modern, cleaner, safer cooking methods and improved nutrition in Kenyan homes. Mediae also produces a series of other educational entertainment media including *Shamba Shape Up*, one of East Africa’s leading agricultural TV shows (https://shambashapeup.com/). *Shamba Chef* uses a reality television format in which a pair of hosts visit a number of guests’ homes and discuss the value of modern cookstoves in the context of other personal and family benefits, such as improved nutrition and the family’s standing in the community. Each TV episode was also adapted for a weekly radio show. Additionally, Mediae developed a digital mobile phone platform called iChef, where viewers could either text or phone for further information, which was not part of the current study. The program attempts to change perceptions of modern cookstove adoption and use based on other social and emotional benefits (e.g., eating and living well, family pride) beyond the stove itself (i.e., to reframe the cookstove in people’s lives), and present those benefits in an entertaining and humorous format [11]. The term “modern stoves and fuels” was used to encompass a range of commercially available energy-efficient wood and charcoal stoves, as well as assorted liquid petroleum gas (LPG) burners and fuel containers. Other fuels and stove types, such as biogas and ethanol, were promoted when applicable.

In 2016, over 85% of households in Kenya still used fuels such as wood, charcoal, and kerosene for their primary cooking fuel [12]. Several recent government initiatives have had an impact on the prices and availability of these fuels and, ultimately, on the way people cook. The forestry ban implemented in 2018, which sought to address deforestation exacerbated by drought conditions, restricted access to freely available woodfuel, which resulted in increased prices for both wood and charcoal [13]. These increases in charcoal prices have pushed more families to use alternative fuels, particularly kerosene or LPG. However, at the same time the Kenyan government accelerated the increase in the excise duty on kerosene [14,15], which had been expected to rise gradually in order to transition consumers progressively to cleaner-burning LPG as that fuel became more available. To fill the emergent need for fuel resulting from the logging ban and rising cost of kerosene, and as a part of synchronized strategies to achieve 35% household adoption of LPG by the year 2030, the Kenyan government simultaneously reduced import taxes on LPG stoves and subsidized the cylinder cost [16]. In addition, the import duty on modern cookstoves was also reduced, in an effort to make these technologies more affordable. However, although the awareness of and aspiration for modern cooking options is high in Kenya, the uptake of these stoves and displacement of traditional technology has to date been limited.

As part of the Alliance’s overall modern cookstove promotion intervention, the authors led an impact evaluation to assess the effects of *Shamba Chef* on modern cookstove adoption outcomes, including knowledge, attitudes, beliefs, intentions, purchase, and changes in modern cookstove use attributed to the program. This study was part of a larger evaluation of four modern cookstove BCC interventions (*Shamba Chef*, another program in Kenya, a program in Bangladesh, and one in Nigeria) described elsewhere [8]. 

The current study sought to answer several broad research questions. These questions framed the study design, measurement, and implementation of data collection. Specifically, we asked:

Was *Shamba Chef* effective in motivating people to purchase and correctly use modern cooking technologies? 

To what degree can the changes in behavior be attributed to *Shamba Chef*? 

Is there a dose–response relationship between higher exposure to *Shamba Chef* programming and the outcomes of positive attitudes, intention to purchase, cookstove purchasing and correct stove usage? 

Were there aspects of *Shamba Chef* that were more effective than others? 

## 2. Materials and Methods 

### 2.1. Intervention

Mediae used a Communication Matrix as its theoretical foundation for *Shamba Chef*. This comprised four questions: What, why, who, and how. What is to be communicated (communication content)? Why is it being communicated and what changes are to be brought about to that effect (communication objectives)? Who is being reached (the audience and communication partners)? And, finally, how is it delivered (which channels of communication)?

*Shamba Chef* had four major themes that ran through the TV series: (1) health implications of current traditional stoves; (2) the savings associated with a modern cook stove; (3) demonstrations of modern cookstoves; (4) the benefits of modern cookstoves, explained by participants on the show. Table 1 summarizes the intervention, including media channels and content.

Thirteen weekly episodes of *Shamba Chef* aired on TV and radio, in English and Swahili. Four episodes featured modern wood burning stoves, seven modern charcoal burning stoves, and two LPG stoves. Running from September 2017 until December 2017, Shamba Chef reached an estimated 5 million people. 

*Shamba Chef* episodes not only showed improved techniques and technologies; it also ran “cook-off” competitions between neighbors and demonstrated how to make highly nutritious and delicious meals, using locally grown produce using improved cooking technologies. The program featured some of Kenya’s top chefs, who would be transported to the rural, on-farm setting and would connect and engage with local women, inspiring them with available and practicable ways of feeding the family. These recommendations were designed to improve the nutritional status of the family members and particularly of children, as well as to increase the adoption of clean cooking technologies. The use and benefits of clean cookstoves were demonstrated and discussed, in order to educate viewers and motivate them to adopt clean cooking.

*Shamba Chef* communicated several key messages through its entertainment education format. These messages attempted to reframe the modern cookstove—or ‘jiko’—in terms of numerous personal, family, social, and emotional benefits. Specifically, *Shamba Chef* communicated the messages shown in Table 2.

### 2.2. Study Design

The underlying evaluation framework was a repeated cross-sectional, quasi-experimental design that assumed levels of BCC exposure will vary by campaign and by medium of exposure. Following previous studies, we anticipated natural variation in exposure to cookstove campaign messages due to the variable resources and efforts of local BCC implementation partners, expenditure of resources, and consumer access to media sources (e.g., mobile phones, mass media) [17,18]. This variation enabled us to create a measure of campaign exposure. We used this measure to calculate a dose–response effect of exposure to *Shamba Chef*, using both self-reported and exogenous measures, similar to an approach developed previously by the investigators [19], which has been successfully applied to large-scale social marketing of behavior change and product adoption in low and middle income countries [19,20]. As a function of higher and lower levels of exposure, we anticipated that the outcomes measured would vary.

### 2.3. Measures/Instruments 

Two types of exposure measures were collected: (1) the self-reported experience of the campaigns, including recognition (by visual aid) and confirmed recall of messages and program-related terminology (e.g., taglines) and images; (2) external independent tracking data from multiple sources to measure potential exposure to cookstove messages at the community, online, and media-market levels.

Self-reported data were collected via household surveys to measure recall and recognition of specific BCC messages delivered by the *Shamba Chef* campaign [21]. Researchers developed a full catalogue of the BCC activities implemented, and questions were asked to capture participation/exposure to each type in the follow up survey, as well as frequency of exposure and reaction/receptivity questions, to assess immediate message response (e.g., was the modern cookstove message credible, likable, shared with friends, or otherwise acted on?). 

The evaluation assessed the effects of the BCC exposures on 7 key outcomes: (1) awareness of modern cookstoves, including biomass and LPG stoves, (2) positive attitudes and knowledge towards modern cookstoves from a visual aid that depicted the stoves and fuels promoted in *Shamba Chef* and available to local consumers in the study catchment area, (3) intention to purchase a modern cookstove within the next month—a visual aid was used to show examples of modern stoves, (4) aspiration to use LPG for cooking, (5) purchase of a modern biomass or LPG stove within the 5 months since the first possible exposure to the BCC, (6) aspiration to own a modern biomass or LPG stove, and (7) aspiration to increase LPG use in the future (for those already using it).

Knowledge was pre-defined, so that the responses to knowledge items could be dichotomized into accurate or inaccurate knowledge. At baseline, sources of current knowledge were also considered, including both sources of current modern cookstove-related knowledge, as well as levels of exposure to selected communication channels, such as TV, radio, etc. 

As watching *Shamba Chef* could have created a community dialogue and increased positive social norms about modern cookstove use, we also asked about diffusion effects (i.e., exposed individuals communicating directly with others about modern cooking options), including conversations the respondent had with community members about cooking, and their reactions and receptivity to these dialogues. 

### 2.4. Data Collection 

Researchers employed household surveys and in-depth interviews (IDIs) to understand the nature and magnitude of the changes, and attribute them where possible to the BCC campaign. The implementation of both qualitative and quantitative evaluation tools allowed for the assessment of the magnitude and frequency of the responses to the BCC campaign, as well as for the exploration of the meaning and understanding of these. A population-based survey was conducted at baseline and at one time point after the *Shamba Chef* show aired. Survey data were collected using mobile data collection technology, ODK (https://opendatakit.org/), with built-in quality and consistency checks. Post-intervention IDIs were also conducted using a series of exploratory open questions. Both the survey and interview were extensively piloted, and the field team was provided with intensive training to ensure that rich accurate information datasets were collected. 

### 2.5. Study Sample

Sample selection was designed to reflect *Shamba Chef*’s target audiences in terms of geography, socio-economic class, age of the cook, and fuel use patterns. Households were selected using a standard approach to avoid any bias or convenience sampling. 

The baseline and post-intervention household surveys combined had a total sample size of 1715 households. Baseline data (N = 855) were collected in February 2017, and post-intervention data (N = 860) were collected in January 2018, shortly after the last episode aired. Post-intervention respondents had had approximately 5 months of potential exposure to the *Shamba Chef* programming.

In all cases, the main participant was the family member who organized the home-keeping, ideally carried out most of the cooking, and was involved in the decision-making for larger household purchases. If the main participant was not involved in the decision-making, the main decision-maker was also interviewed. 

A total of 141 IDIs were conducted with four groups of participants: purchasers, non-purchasers, exposed, and unexposed. Participants for the IDIs were drawn from either the survey sample or sales lists provided by distributors of the promoted modern stoves in the study areas, depending on the study group and location.

The study protocol was approved by the institutional review board (IRB) at Advarra (https://www.advarra.com/ protocol number Pro00022033). Local permission was also sought and secured. Informed and voluntary consent was obtained from all study participants for all data collection methods, including permission to take and use photographs. 

### 2.6. Data Analysis

All survey data were analyzed in SAS 9.4 (Cary, NC, USA). Descriptive statistics summarized frequencies, percentages, means, and standard deviations (SD) for socioeconomic data, demographics, stove behaviors, media use, and key variables for *Shamba Chef* exposures and outcomes. 

To model the dose–response effects of *Shamba Chef* exposure, we developed several variables/indices: (1) a summed exposure to all *Shamba Chef* BCC materials, based on self-reported frequency of seeing/hearing/experiencing each one; (2) individual BCC *Shamba Chef* materials delivered on TV and radio as their own exposure; (3) a sum of all reported sources of exposure for knowing about modern biomass stoves (e.g., friends, family, radio, TV, webisode, etc.). Multivariable logistic regression models assessed the impact of each exposure on each dichotomous outcome, while adjusting for potentially relevant covariates, such as age, sex, education, and socioeconomic status, as well as other variables that could be related to the outcomes and exposures, such as stove use and media use. Covariates that were potentially associated with the outcomes and exposures were assessed in crude (i.e., unadjusted) analyses. Covariates with suggestive evidence of associations with the outcomes (i.e., *p*-value ≤0.10) were included in full logistic regression models to adjust for their potential effects. Reduced model iterations were explored by removing the covariates with the highest p-values and least impact on the model, while assessing changes in the effect and precision of the exposure on the outcome. Final summaries of the adjusted logistic regression models for each outcome were reported as odds ratios (OR) and 95% confidence intervals (CI).

For the IDI data, thematic analysis was carried out using NVivo 12 qualitative analysis software (QSR International, 2018), to synthesize and interpret all qualitative data. The transcripts of the IDIs were initially reviewed by two members of the research team and a provisional coding frame was created based on the research questions and themes of interest. Nvivo codes were added as unanticipated themes were introduced. Coding frames were continually reviewed by the analysis team for duplication and refined accordingly. 

## 3. Results

### 3.1. Quantitative Results 

The post-intervention sample included participants from urban (39%), peri-urban (47%), and rural areas (14%) (Table 3). The majority of participants were married females, between the ages of 23 and 50, and had completed some secondary school education or higher. Just over 60% of participants had paid work outside the home at endline. In keeping with the *Shamba Chef* target audience, all households were within the lower to lower-middle-income socio-economic class, as defined by the living standards measure (LSM). The LSM divides the population in to 17 LSM groups, 17 (highest) to 1 (lowest). The average number of people eating an evening meal in the household, excluding infants, was 3.6 (SD 2) at both time points. Note that the sample sizes for baseline and follow up were repeated cross-sections and represent distinct enrollments.

Table 4 summarizes self-reported exposure to *Shamba Chef* TV and radio broadcasts. A representative clip of the TV and radio versions of the show were used as visual (J) and audio (M) aids. For analysis purposes we dichotomized the data into those exposed one or more times compared to never. Data were captured only at the post-intervention stage when the audience had potentially seen or heard the program on TV or radio.

The relationship between exposure and the seven outcomes of interest were examined using multivariate models (Table 5). In brief, exposure to visual aid J (TV) and audio aid M (radio) had a strong impact, independent of other factors, on awareness of modern biomass stoves (OR 4.4; 95% CI 2.8 to 6.9), and aspirations to own a modern biomass stove (OR 2.0; 95% CI 1.4 to 2.9) compared to those with no exposure. 

Exposure to visual aid J on TV seemed to have a stronger influence than the radio audio aid M. Receiving information about modern stoves from two or more sources generated greater awareness of LPG stoves (OR 2.0; 95% CI 1.3 to 3.1). No other outcomes were influenced by the main exposures (Table 6).

### 3.2. Qualitative Results

We identified a number of major themes in the 141 IDIs. These include cookstove awareness, information sources, perceptions, barriers to uptake, gender roles, and evidence of *Shamba Chef* impact.

**Awareness of promoted cookstoves.** The level of awareness of modern biomass stoves was high, with widespread positive attitudes. General knowledge of the potential benefits was also high; however, the modern biomass stoves were less-often reported to be an aspirational stove than LPG. Use, awareness, and desirability of LPG was widespread. However, perceptions of LPG stoves were negatively influenced by anxiety over possible safety hazards. 

**Sources of information on clean cooking.** According to all participant groups—exposed, non-exposed, purchasers, and non-purchasers—there are many sources from which information about improved cookstoves can originate. For example, participants reported that they saw friends and family members using certain improved stove types, such as LPG, and that this increased their aspiration to own one themselves. Some participants reported taking part in women’s groups or chamas, where the use of modern cookstoves was discussed and generally viewed positively and as a sign of success. Additionally, most participants reported seeing TV and radio advertisements for various modern cookstove brands and models, as well as having encountered sales representatives traveling door-to-door or stationed in supermarkets, offering information on modern cookstoves and payment plans.

**Perceptions of improved stoves.** Due to the several sources of information available to the survey respondents, many respondents were familiar with and had positive perceptions of modern stoves—both biomass and LPG. The positive perceptions cited most often by interview participants included fuel efficiency and subsequent savings of time and money for fuel procurement; cleanliness of food, kitchenware, and kitchens; convenience in terms of faster cooking and a generally elevated standard of living. Of the few negative themes that emerged, participants noted that the initial cost of the stoves can be high, and that changing methods and costs for fuel procurement can present a barrier.

**Barriers to stove uptake.** Overall, new cookstoves are competing against other household items in liquidity-constrained homes. For modern biomass stoves, the key barriers were: (1) initial cost of the stove; (2) value—although many recognize the benefits of the modern biomass stoves, they are not viewed as a greater value proposition than traditional stoves; (3) lack of information on specific stoves; (4) increases in the price of charcoal and decreases in reliable sources of wood fuel leading more people to consider gas. For LPG, the initial outlay was reported to be the main barrier, although the fear of explosion was particularly acute in households with younger children. 

**Gender roles in stove purchase.** Many women reported that they alone make the purchase decision; spousal permission seemed to be a requirement in a minority of cases. It was usually the women who chose the stove type and made the purchase. When men did become involved, they primarily contributed financially: “… he topped up the amount I had saved.” When a woman had paid work outside the home or her own business, she was more likely to make independent decisions.

**Impacts of the Mediae BCC campaign.** Many of the exposed purchasers described how *Shamba Chef* influenced their decision to purchase a modern biomass stove. *Shamba Chef* served to clearly explain how the stoves work, outline and confirm their purported benefits, and engage with real people to encourage participants to trust and to purchase those cookstoves. Many were actively considering purchase prior to watching the show, motivated by the advertised faster cooking and fuel savings. Seeing the stove used on the show provided the affirmation of its benefits that was needed to trigger purchase. The women also felt reassured and empowered by the simple clear information on how to use the stoves.

Overall, *Shamba Chef* was more likely to motivate the purchase of a modern biomass stove than of the more ubiquitous gas. It was most influential with people who relied solely on traditional cooking methods, but also occasionally had role in persuading LPG owners to consider purchasing a modern biomass stove. Possible reasons for the relative strength of the biomass messaging include: only two out of thirteen episodes featured gas stoves, LPG benefits are already well established, and the main barrier to LPG is liquidity constraints—rather than lack of awareness or aspiration.

Exposed non-purchasers reported similar impacts of exposure to *Shamba Chef* as those who purchased, but they were overwhelmingly prevented from purchasing a modern cookstove due to lack of financial stability. 

## 4. Discussion

*Shamba Chef* represents a novel approach to clean cooking promotion. The use of educational entertainment and a reality TV format has not been previously attempted in the cookstove sector, and—given the success of similar approaches in other fields—such as sexual health and family planning, it is a promising strategy [22,23,24]. *Shamba Chef* ran for only one season and operated in an environment with significant barriers to produce behavior change, but, despite these limitations, achieved success in promoting demand for modern cookstoves. 

With respect to our first research question—whether *Shamba Chef* motivated people to purchase and correctly use cleaning cooking technologies—results were mixed. The quantitative evaluation showed that exposure to the programming increased aspiration to purchase a modern cookstove (both improved biomass and LPG stoves), and increased intention to purchase. We did not see an effect of exposure on purchase. The latter may be explained by a lack of financial resources and, in some situations, availability of stoves to the survey respondents.

Qualitative results show that exposed purchasers were highly motivated by the *Shamba Chef* programming, that it increased their aspiration to purchase a modern biomass stove, and that their purchase decision was, at least in part, driven by messages and content presented in the program. Exposed non-purchasers also showed increased aspiration, but financial constraints represented a major barrier to purchase. The combined quantitative and qualitative results suggest that *Shamba Chef* was educational and persuaded many participants to seek a clean cooking solution, but audience segmentation moderated these effects. Future research should examine these interactions between ability and intention or willingness to purchase in depth.

With respect to the question of attribution, we see that exposure was associated with increased awareness of modern cookstoves, aspirations to purchase both modern biomass and LPG stoves, and intentions to purchase. We also see evidence of a dose–response relationship in that exposure to *Shamba Chef* from two or more sources was associated with increased awareness of modern cookstoves and increased intentions to purchase. Thus, repeated exposure over time may be an effective demand creation strategy, as shown in previous studies [19,25], and should be explored further, through additional seasons of programming like *Shamba Chef*, and through future research. 

With respect to which parts of *Shamba Chef* may have been the most effective, the quantitative evaluation demonstrated that two specific visual/audio aids that presented scenes from the program, their messages and their content were associated with observed increases in awareness, aspiration to purchase, and intention to purchase. The qualitative evaluation provides greater insight into this question. Specifically, the functional (ease and speed of cooking) and social/emotional (sense of modernity and living well) benefits were associated not only with improved attitudes and intentions related to modern cookstoves, but were also drivers of purchase decisions. This effect is consistent with the literature on the successful branding of behavior change interventions [26]. The educational entertainment strategy, and its specific promise of benefits to viewers, appears to have been successful in motivating behavior change based on the qualitative findings. These benefit perceptions should be explored in greater detail, and the extent to which they can create sufficient demand to offset barriers to purchase, such as cost, should be investigated in future research and programming [27]. 

Given these findings, *Shamba Chef* has important implications for demand creation efforts in the cookstove sector. Clearly, educational entertainment is a promising strategy. The quantitative study showed that exposure to the messages and content produced attributable, positive effects on short-term modern cooking-related outcomes, including the intention to purchase. In an environment that addresses the economic barriers to purchase, including through subsidies or other incentives, the *Shamba Chef* approach may lead to increased purchase. Future studies should include controlled experiments on how to create conditions with reduced purchase barriers in order to evaluate the possible dose–response effects of such programming on the actual purchase and use of modern cookstoves.

The qualitative study provided valuable insights into how *Shamba Chef* created demand. The benefits communicated in the programming, which sought to shift perceptions of modern cooking and highlight how it can improve a family’s lifestyle, were clearly received by exposed individuals. *Shamba Chef* depicted modern cooking as socially desirable and part of being a successful, contemporary family in Kenya. According to the social norms literature, creating a positive, normative belief in modern cooking may promote adoption [28,29]. Previous studies on cookstoves suggest peer norms may predict adoption [30]. Future research should examine how to refine and enhance norms-based messages and content in order to maximize effects on demand—particularly in light of specific challenges related to cooking energy transitions such as stove stacking and adoption niches [31].

Despite the overall success of *Shamba Chef* and findings of this research, the project had some limitations. First, funding was only made available for one season of the program (after initially being planned for two seasons), which limited the research team’s ability to examine longer-term effects and may have contributed to the lack of observable quantifiable effects of program exposure on purchase and use. Second, the study was cross-sectional; therefore, we cannot make specific attributions of exposure effects on a cohort of respondents over time. Third, we are aware that Kenya has had and continues to have numerous commercial brand promotions for modern cookstoves. The potential confounding effects of these other commercial campaigns was not quantified in the current research and may be an important co-variate in future studies.

Future research should follow a longitudinal cohort with additional cross-sectional samples to achieve both population-level tracking and to measure change over time. Using an experimental design that deploys various supply and cost incentives to reduce barriers to purchase, researchers could compare BCC approaches and isolate the effects of a program such as *Shamba Chef* on purchase and use over time. The confounding effects of these barriers, as well as other commercial cookstove promotion efforts, should be addressed in future research designs.

## 5. Conclusions

This study demonstrated that the *Shamba Chef* educational entertainment TV and radio series was effective in promoting determinants of modern cookstove demand. The qualitative research helped to explain this increased demand through greater aspirations and perceived benefits of clean cooking in part through exposure to *Shamba Chef* messages.

## Figures and Tables

**Table 1:** Summary of *Shamba Chef* intervention.

Location	Aim	BCC Media Channels and Themes	Dates	Estimated Reach
Kenya. Urban, peri-urban and rural areas.	To increase knowledge and awareness of the benefits of modern cookstoves. To educate on where to buy modern cookstoves and how to finance them. To promote improved nutrition for the family.	The Mediae campaign featured the *Shamba Chef* TV and radio show, which focused on modern cooking options and nutrition. Thirteen episodes promoted a range of fuels and technologies, and featured home makeovers and competitions. In addition to connecting through social media, viewers could also subscribe to a free interactive mobile platform called iChef to access more information.	Aired once/week for 60 minutes from Sept-Dec 2017	About 5 million people

**Table 2:** *Shamba Chef* Message Content.

Modern Cookstoves	Nutrition	Taglines
Save money and save time in the kitchen. Reduced smoke—means a cleaner kitchen, no more coughing, or watery eyes. Uses little charcoal. Cook fast and efficiently. It is an investment, which lasts 1–5 years. Stoves have a 1–2-year warranty and can be repaired or replaced for free. You can buy with loans through chamas, or saccos *. You can connect to iChef for information on how to buy. It is a modern solution for a modern kitchen. A modern man buys a modern cookstove to take care of his family.	A balanced diet is important for the family’s health. There are five groups of foods essential for family health. We should eat a food from these five groups every day: Animal Protein Plant Proteins Vegetables Fruits Starchy Staples. The first 1000 days are the most important in a child’s life for growth and development. Good nutrition is important for a child to grow healthy and strong. Good nutrition is the best thing for a child’s future.	We explore new ways to cook, to make it quicker, safer, cleaner and at half the cost The families, chefs and neighbors compete to make “the most delicious and most nutritious meal”.

* Chamas and Saccos are informal self-help cooperatives that are normally used to pool and invest savings by its members.

**Table 3:** Demographic and Socioeconomic Data.

		Baseline (N = 855)		Post-Intervention (N = 860)	
**Sex of main respondent**		**Count**	Percent (%)	Count	Percent (%)
1	Male	78	9	117	14
2	Female	776	91	743	86
**Sex of decision-makers in household – if different from the main cook**					
1	Male	10	45	10	91
2	Female	12	55	1	9
Groups too small to perform chi-square					
**Education of the respondent**					
1	None, primary incomplete, primary complete	360	42	326	38
2	Some secondary or higher	495	58	532	62
**Education of the primary earner**					
1	None, primary incomplete, primary complete	443	52	456	53
2	Some secondary or higher	408	48	401	47
**Marital status**					
1	Married	605	71	559	65
2	Single, separated, divorced, widowed, living together	250	29	297	35
**Paid work outside the home**					
1	Yes	480	56	542	63
2	No	375	44	318	37

**Table 4:** Exposure to Shamba Chef TV and radio broadcasts at post-test.

Variable	Response	Freq.	Percent (%)
**How frequently have you seen visual aid J before today- *Shamba Chef* TV?**			
1	One or more times	311	47
2	Never	354	53
**How frequently have you heard audio aid M before today- *Shamba Chef* radio?**			
1	One or more times	225	30
2	Never	521	70

**Table 5:** Relationship between exposure and the seven outcomes of interest.

Multivariable (Adjusted) Model: (Number of Observations Used); Odds Ratio (95% CI)					
Exposures of Interest	Awareness of Improved Biomass Stoves^1^	Awareness of liquefied petroleum gas (LPG) Stoves^1^	Positive Attitudes as a Dichotomous Variable ^1,3^ *Filtered to Those Who were Aware of Modern Cookstoves before Survey*	Intention to Purchase a Modern Stove within Next Month^1^ *Filtered to those who were Aware of Modern Cookstoves before Survey*	Aspiration to Use LPG for Cooking among those Who do not Currently own LPG^1^ *Filtered to those that Said ‘no’ to LPG Ownership.*
**1.** Dichotomous variable: total number of exposures to combined visual and audio aids J and M 1 one or more times ^2^ 2 never (ref)	(N=738) 4.4 (2.8 to 6.9)	(N=738) 1.3 (0.9 to 1.9)	(N=600) 0.7 (0.4 to 1.0)	(N=601) 1.5 (0.8 to 2.9)	(N=342) 1.1 (0.6 to 2.3)
**2.** Frequency of exposure to individual BCC visual and audio aids J and M ^2^					
**2a.** Visual aid J: Shamba Chef TV 1 one or more times ^2^ 2 never (ref)	(N=658) 3.2 (1.5 to 6.9)	(N=657) 1.2 (0.8 to 1.9)	(N=598) 0.5 (0.4 to 0.8)	(N=599) 1.2 (0.7 to 2.3)	(N=295) 0.9 (0.4 to 1.9)
**2b.** Audio aid M: Shamba Chef Radio 1 one or more times ^2^ 2 never (ref)	(N=738) 1.5 (0.9 to 2.4)	(N=738) 1.2 (0.8 to 1.8)	(N=600) 1.4 (0.9 to 2.2)	(N=601) 1.1 (0.6 to 2.0)	(N=342) 1.2 (0.6 to 2.5)
**3.** Sum of exposure to all possible sources of information related to new stove marketing. *Filtered to those said “yes” to the question, “F1. Before this survey had you heard, seen or been told any information about new cookstoves such as these (visual aid A).* **Dichotomized: Sum of all reported sources of exposure** 1 source of exposure (reference) 2 or more sources of exposure	All responses have same response due to filtering based on the outcome of interest.	(N=601) 2.0 (1.3 to 3.1)	(N=599) 1.1 (0.7 to 1.6)	(N=1162) 0.6 (0.4 to 0.8)	(N=248) 1.1 (0.5 to 2.4)

**Table 6:** Relationship between exposure and the seven outcomes of interest (continued).

Multivariable (Adjusted) Model: (Number of Observations Used); Odds Ratio (95% CI, *p*-Value)				
Exposures of interest	Purchase of an LPG stove within the BCC exposure period^1^ *Filtered to those who had bought an LPG stove*	Aspirations to own a modern biomass stove^1^	Aspirations to own an LPG stove^1^	Aspirations to use more LPG more than you do now ^1^ *Filtered to those who said ‘yes’ to LPG ownership*
**1.** Dichotomous variable: total number of exposures to combined visual and audio aids J and M 1 one or more times ^2^ 2 never (ref)	(N = 176) 1.2 (0.4 to 3.4)	(N = 724) 2.0 (1.4 to 2.9)	(N = 724) 0.7 (0.5 to 0.9)	(N = 229) 0.6 (0.3 to 1.2)
**2.** Frequency of exposure to individual BCC visual and audio aids J and M ^2^				
2a. Visual aid J: Shamba Chef TV 1 one or more times ^2^ 2 never (ref)	(N = 156) 0.7 (0.2 to 1.9)	(N = 643) 1.6 (1.1 to 2.3)	(N = 643) 0.7 (0.5 to 1.0)	(N = 197) 0.6 (0.3 to 1.2)
2b. Audio aid M: Shamba Chef Radio 1 one or more times ^2^ 2 never (ref)	(N = 176) 1.6 (1.7 to 18.8)	(N = 724) 1.5 (1.1 to 2.2)	(N = 724) 0.8 (0.6 to 1.2)	(N = 229) 1.0 (0.5 to 2.0)
**3.** Sum of exposure to all possible sources of information related to new stove marketing. *Filtered to those said “yes” to the question, “F1. Before this survey had you heard, seen or been told any information about new cookstoves such as these (visual aid A).* **Dichotomized: Sum of all reported sources of exposure** 1 source of exposure (reference) 2 or more sources of exposure	(N = 150) 0.5 (0.1 to 1.6)	(N = 1144) 1.1 (0.8 to 1.4)	(N = 1144) 0.9 (0.7 to 1.2)	(N = 188) 0.7 (0.3 to 1.4)

^1^ Full models were adjusted for the exposure of interest plus 12 covariates: socioeconomic status (LSM score), area (urban, peri-urban, or rural), education, age, sex, marital status (married vs. all else), use of TV, radio, internet, mobile phone, and social media, and ownership of two or more stove types. These covariates were selected based on their associations with the outcome of interest. The adjusted effects of the exposure on the outcome did not vary in effect measure or precision based on inclusion or removal of these covariates; therefore, the full models are presented. ^2^ Exposure data only available from the endline survey, question not asked in baseline. ^3^ Attitudes towards modern cookstoves stoves included 12 items: less fuel, saves money, saves time, reduces smoke, cleaner kitchen, accessible, warranty for years, reduces health issues, loans are available, solution for a modern kitchen, male can purchase, overall quicker/safer/cleaner/cheaper.
